# Association between paternal smoking at the time of pregnancy and the semen quality in sons

**DOI:** 10.1371/journal.pone.0207221

**Published:** 2018-11-21

**Authors:** Jonatan Axelsson, Sally Sabra, Lars Rylander, Anna Rignell-Hydbom, Christian H. Lindh, Aleksander Giwercman

**Affiliations:** 1 Molecular Reproductive Medicine, Department of Translational Medicine, Faculty of Medicine, Lund University, Malmö, Sweden; 2 Occupational and Environmental Medicine, Laboratory Medicine, Faculty of Medicine, Lund University, Lund, Sweden; Universite Clermont Auvergne, FRANCE

## Abstract

**Background:**

Maternal smoking during pregnancy has repeatedly been associated with decreased sperm counts in sons. Nevertheless, our team recently detected a lower total sperm count in the sons of smoking fathers as compared to sons of non-smoking fathers. Since paternal and maternal tobacco smoking often coincide, it is difficult to discriminate whether effects are mediated paternally or maternally when using questionnaire- or register-based studies. Therefore, getting an objective measure of the maternal nicotine exposure level during pregnancy might help disentangling the impact of paternally and maternally derived exposure.

**Objectives:**

Our aim was to study how paternal smoking at the time of the pregnancy was associated with semen quality in the sons after adjusting for the maternal levels of nicotine exposure during pregnancy.

**Methods:**

We recruited 104 men (17–20 years old) from the general Swedish population. The participants answered a questionnaire about paternal smoking. Associations between smoking and semen volume, total sperm count, sperm concentration, morphology and motility were adjusted for levels of the nicotine metabolite cotinine in stored maternal serum samples obtained from rubella screening between the 6^th^ and 35^th^ week of pregnancy. We additionally adjusted for the estimated socioeconomic status.

**Results:**

After adjusting for the maternal cotinine, the men of smoking fathers had 41% lower sperm concentration and 51% lower total sperm count than the men of non-smoking fathers (p = 0.02 and 0.003, respectively). This was robust to the additional adjustment.

**Conclusions:**

Our results suggest a negative association between paternal smoking and sperm counts in the sons, independent of the level maternal nicotine exposure during the pregnancy.

## Introduction

Maternal smoking during pregnancy has been associated with reduced sperm concentration in the sons in several studies [1–6], which is not the case for paternal smoking [2–5].

However, in a study of young Swedish men, we detected that paternal smoking at the time of the pregnancy was negatively associated with both sperm concentration and the total sperm count [7]. Although this cannot be excluded as a chance finding, paternal smoking around pregnancy has been associated with other negative outcomes in children, such as a shorter reproductive life-span in daughters [8], malformations [9], and childhood leukaemia [10]. Since smoking can damage sperm DNA [9] the findings above might indicate that paternal smoking prior to the time of the conception could negatively affect the offspring’s health, maybe due to mutations or epigenetic alterations in the male germ cells [9, 11].

Nevertheless, up to now, studies on parental smoking and sons’ semen quality have been based on data from questionnaires or registers which may somewhat underestimate the impact of maternal smoking [12, 13] and not fully mirror the actual dose of the maternally derived exposure to tobacco smoke. Further, maternal and paternal smoking is reported to often coincide [14]. Therefore, potential effects of these two different exposures may be difficult to disentangle [11]. Since we in our previous study, in line with the other mentioned studies on maternal smoking and son’s semen quality [1–6], noted that maternal smoking was associated with a decreased sperm concentration [7], we could not exclude that the association between paternal smoking and the lower sperm counts nonetheless was rather due to maternal smoking that was never reported to the Medical Birth Register, or due to maternal secondary tobacco smoke exposure, the information on which could not be derived from questionnaires or the Medical Birth Register. Moreover, smoking is associated with a low socioeconomic status [15, 16], which also has been associated with lower sperm counts [17]. Thus, we could neither exclude that the association between paternal smoking and lower sperm counts in the sons was rather due to a low socioeconomic status. These two problems could be alleviated by adjusting for the actual level of maternal exposure to nicotine, measured in a biological matrix from the mother during a relevant period of time, as well as by trying to additionally adjust for the socioeconomic position.

Nicotine exposure can be quantified by assessing the levels of the metabolite cotinine in biological fluids [18]. Those levels are closely correlated with the number of cigarettes smoked [19] and objectively reflect the body’s internal dose of exposure [20, 21]. Levels of cotinine also seem to be more closely related to birth outcomes than does the reported number of smoked cigarettes per day [22].

Through a previous study on prenatal exposure to phthalates [23], we had access to levels of cotinine in maternal serum obtained during the pregnancy, as well as to data on paternal smoking at the time of the pregnancy, in 104 men from the general Swedish population. In addition, we had access to data on the maternal occupation during the pregnancy. Therefore, we were now able to study associations between paternal smoking at the time of the pregnancy and semen quality in the sons, while adjusting for both the pregnancy-based actual dose of maternal exposure to nicotine and the socioeconomic status, based on the maternal occupation.

## Materials and methods

**Fig 1:**
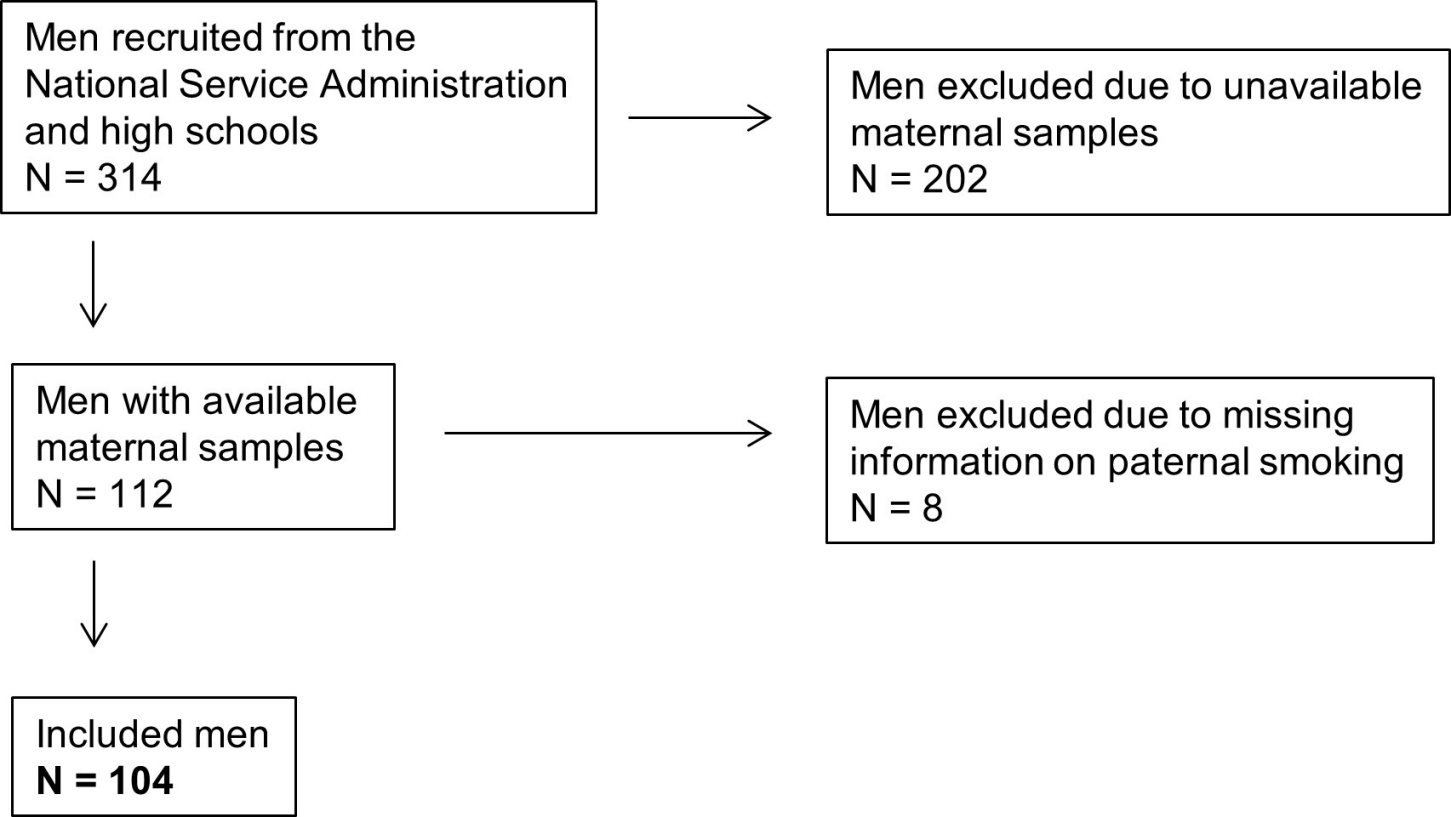
Flow chart describing the inclusion of the study subjects.

From 2008 to 2010 we recruited 314 men who were 17–20 years old. The men were recruited through the Swedish military service administration and from high schools for a study of male reproductive function [24]. For inclusion, the men and their mothers had to be born and raised in Sweden, and the men had to live within 60 km from the city of Malmö in Southern Sweden. The study was approved by the regional ethical review board, “Regionala etikprövningsnämnden i Lund” according to protocol “2008/5” and case/registration number “2008/181”. The men signed an informed consent and filled in questionnaires regarding current own smoking, paternal smoking during the pregnancy (yes or no) as well as regarding the mother’s occupation during the pregnancy. They underwent a genital examination by a physician, which included the presence of varicoceles. Finally, all the recruited men delivered a semen sample. We were able to retrieve 112 maternal serum samples of our male participants. These samples were drawn for rubella screening between the 6^th^ and the 35^th^ week (mean 12 weeks) of pregnancy [23]. The samples had been stored in a biobank at -80°C. There was no difference present in the semen quality between these 112 men and the 202 men of whom there were no available maternal samples (data not shown). Through the questionnaires filled in by the recruited men, data on whether the father had smoked or not during the pregnancy was available for 104 of the 112 men with available maternal samples. These 104 men were the ones included in this study (Fig 1).

Among the 104 men, 25 men (24%) were smokers, and 79 men (76%) were non-smokers. The smokers had an average of 6.6 cigarettes smoked per day, and had smoked for a mean duration of 2.9 years. Thirty-three men (32%) reported that their father smoked during the pregnancy, and 71 men (68%) that their father had not. No additional information was available regarding the paternal smoking through the questionnaires. Among the men with smoking fathers, 12% had a varicocele at the physical examination, whereas 11% had a varicocele among the men with non-smoking fathers. This difference was not statistically significant.

Based on the reported maternal occupation during the pregnancy, which was either missing or not classifiable in 33 men, we estimated the maternal socioeconomic status of the remaining 71 men. The maternal occupations were categorised according to the socio-economic classification system of Statistics Sweden [25] into five categories, from unskilled and semiskilled workers (12 mothers), skilled workers (11 mothers), assistant non-manual employees (13 mothers), intermediate non-manual employees (27 mothers), to employed and self-employed professionals (8 mothers). No statistically significant differences in the variables of semen quality were present between the 71 men for whom we could estimate the maternal socioeconomic status and the 33 men with mothers that we could not classify.

### Analysis in maternal serum samples

Cotinine was analysed by liquid chromatography-tandem mass spectrometry as described previously [23]. In brief, 100 μL of serum was added with an isotopically labelled standard, and samples digested with glucuronidase. Acetonitrile was used to precipitate proteins. The samples were thereafter analysed by use of a triple quadrupole linear ion trap mass spectrometer, coupled to a liquid chromatography system, in a negative ion mode. The coefficient of variation was 2% at 48 ng/mL, and the limit of detection (LOD) was calculated to 0.4 ng/mL.

### Semen analysis

The men were asked to keep 48–72 hours of sexual abstinence, a requirement which was fulfilled by 42% of the men, but all men except one had their actual time of abstinence recorded.

The semen samples were delivered after masturbation at the hospital, and were examined regarding volume, sperm concentration, total sperm count, as well as regarding the proportions of morphologically normal and progressively motile sperm. All analyses were done according to the–at that time–most recent WHO guidelines [26]. Data on sperm concentration and total sperm count were missing in one man. This participant delivered a sample with a semen volume of 0.10 mL, and reported spillage of the sample during collection.

### Statistical analysis

SPSS version 22 was used for statistical calculations, and p < 0.05 was considered statistically significant.

For background information, all variables were used untransformed (Table 1).

**Table 1:** Background information overall and according to paternal smoking, untransformed variables.

	All					Paternal smoking	
						Yes (n = 33)	No (n = 71)
	N	Minimum	Maximum	Median	Mean ± SD	Mean	Mean
Age (years)	104	17.5	20.5	18.3	18.3 ± 0.41	18.4	18.3
BMI (kg/m^2^)	104	18	37	22	23 ± 3.3	23	23
Foetal age at sampling (days)	104	39	240	81	86 ± 33	89	84
Maternal age at sampling (years)	104	17	41	27	28 ± 5.3	29	27
Maternal cotinine (ng/mL)	104	0.06	250	0.74	36 ± 64	62	24
Sperm concentration (million/mL)	103	0.3	300	53	69 ± 60	52	77
Total sperm count (million)	103	1	1400	140	200 ± 210	130	230
Semen volume (mL)	104	0.10	9.1	2.7	2.8 ± 1.4	2.7	2.8
Morphologically normal (%)	104	0.0	21	9.0	8.7 ± 5.7	7.6	9.2
Progressive sperm (%)	104	2.0	86	58	53 ± 17	50	54

Abbreviations: SD, standard deviation

For statistical calculations, sperm concentration and total sperm count were transformed by the cubic root [27], which gave a better distribution of residuals. Further, the foetal age in days at the maternal sampling was transformed by the natural logarithm for a more normal distribution, all to possibly increase the statistical prediction [28]. The 18 values of cotinine below LOD (17%) were used unedited, as recommended by the Analytical Methods Committee of the Royal Society of Chemistry [29]. However, for a better distribution of residuals, all the levels of cotinine were transformed by the natural logarithm.

We controlled improvements in distributions through the values of skewness and kurtosis, and performed tests of normality as well as histograms and plots, all to assess the residuals’ linearity, normality and scedasticity.

Associations between paternal smoking (yes/no) and the parameters of semen quality were studied by use of general linear models, primarily unadjusted, and subsequently adjusted by the simultaneous inclusion of maternal level of cotinine (as a continuous variable) and the other potential confounders: own smoking (yes/no), body mass index (BMI), days of foetal age at birth and maternal age at sampling (years), as well as the time of abstinence (hours). These potential confounders were chosen based on what we considered biologically plausible to possibly confound an association between paternal smoking and the semen quality of the sons.

Thereafter, the calculations above were repeated by use of cotinine as a categorised variable. Cotinine levels were categorised as: unexposed if below 0.4 ng/mL (18 men); environmentally exposed for levels ranging from 0.4 ng/mL to < 15 ng/mL (57 men); and as smoking for cotinine levels at 15 ng/mL or higher (29 men). This categorisation is similar to that applied in a previous study [13], although we used our somewhat higher LOD (0.4 ng/mL) as the cut-off between unexposed and environmentally exposed, instead of 0.2 ng/mL which was the LOD in the other study [13].

Moreover, since both 15 ng/mL [13] and 5.3 ng/mL [19] have been suggested as cut-offs for daily active smoking during pregnancy, we also repeated the analysis using ≥ 5.3 ng/mL instead of ≥ 15 ng/mL as the cut-off to distinguish smoking mothers from non-smoking mothers.

Since a low socioeconomic status has been associated with both smoking during pregnancy [15] and a decreased semen quality [17], and therefore could not be excluded as an additional potential confounder, we recalculated the statistically significant estimates of the analyses in which we used cotinine as a continuous variable by inclusion of an additional adjustment for the estimated maternal socioeconomic status in the 71 men with available data.

We calculated the so called variance inflation factor through general linear models with paternal smoking as the dependent variable and the other potential confounders as independent variables. This resulted in a variance inflation factor of 1.2 without socioeconomic status and 1.3 with socioeconomic status included. This indicates that no problem of collinearity was present [30] in the statistical analyses.

Finally, as a comparison with our models adjusted for the potential confounders, we stratified the data without any adjustments, but by excluding men that had mothers categorised as smokers (cotinine ≥ 15 ng/mL), after which we studied differences in semen parameters between the men with smoking and non-smoking fathers.

## Results

Characteristics of the participants, including untransformed semen parameters and maternal levels of cotinine, are shown in Table 1.

Paternal smoking was, unadjusted, negatively associated with both sperm concentration (p = 0.02) and total sperm count (p = 0.02), with similar findings after adjustment for maternal levels of cotinine and the other potential confounders (p = 0.02 and 0.003, respectively; Tables 2 and 3).

**Table 2:** Regression coefficients [95% CI] between paternal smoking and semen parameters.

		Sperm concentration^a^	Total sperm count^a^	Semen volume (mL)	Normal morphology (%)	Progressive motility (%)
Paternal smoking (yes vs no)	Unadjusted	-0.58 [-1.1, -0.09]*	-0.96 [-1.7, -0.18]*	-0.13 [-0.70, 0.43]	-1.5 [-3.9,0.85]	-3.4 [-10, 3.6]
	Adjusted^b^	-0.64 [-1.2, -0.13]*	-1.2 [-2.0, -0.42]**	-0.37 [-0.93, 0.19]	-1.7 [-4.4, 0.90]	-3.1 [-11, 4.6]

* P < 0.05

** P < 0.01

^a^ transformed by the cubic root

^b^ adjusted for age, BMI, own smoking, maternal age, foetal age at sampling, abstinence time and continuous levels of maternal cotinine

**Table 3:** Mean values in semen parameters according to paternal smoking at the time of the pregnancy.

		Father smoker	Father non-smoker	P
**Sperm concentration (million/mL)**^**a**^	Unadjusted	**38**	**62**	**0.02**
	Adjusted^b^	**38**	**64**	**0.02**
	Adjusted^c^	**36**	**61**	**0.02**
	Adjusted^d^	**39**	**72**	**0.03**
**Total sperm count (million)**^**a**^	Unadjusted	**92**	**160**	**0.02**
	Adjusted^b^	**88**	**180**	**0.003**
	Adjusted^c^	**90**	**170**	**0.006**
	Adjusted^d^	**92**	**210**	**0.01**
**Semen volume (mL)**	Unadjusted	2.7	2.8	0.64
	Adjusted^b^	2.5	2.9	0.19
	Adjusted^c^	2.8	3.0	0.34
**Normal morphology (%)**	Unadjusted	7.6	9.2	0.20
	Adjusted^b^	7.2	8.9	0.19
	Adjusted^c^	6.7	8.6	0.14
**Progressively motile (%)**	Unadjusted	50	54	0.34
	Adjusted^b^	51	54	0.42
	Adjusted^c^	48	52	0.31

^a^ back-transformed from the cubic root

^b^ adjusted for BMI, own smoking, maternal cotinine as a continuous variable, foetal age, maternal age and abstinence time

^c^ like ^b^ but with maternal cotinine categorized as < 0.4 ng/mL (unexposed), 0.4 to < 15 ng/mL (environmentally exposed) or ≥ 15 ng/mL (smoker)

^d^ like ^b^ but additionally adjusted for maternal socioeconomic status

Thus, compared with men of non-smoking fathers, the men of smoking fathers had 41% lower sperm concentration (38 x 10^6^/mL vs. 64 10^6^/mL) and 51% lower total sperm count (88 x 10^6^ vs. 180 x 10^6^) after the adjustments (Table 3). Similar findings were done when we used cotinine as a categorised variable (Table 3). When we repeated the analyses using the lower cut-off of ≥ 5.3 ng/mL for cotinine/mL instead of ≥ 15 ng/mL to identify smokers, this resulted in two additional mothers categorised as smokers but the above mentioned statistically significant associations remained significant, with no additional ones appearing (data not shown).

When we additionally adjusted for the maternal socioeconomic status, none of the differences in sperm numbers between men of smoking fathers and men of non-smoking fathers lost their statistical significance (Table 3), and none of the regression coefficients were weakened by more than 10% (data not shown).

In the stratified model in which we excluded men having mothers who were categorised as smokers (cotinine ≥ 15 ng/L), a similar picture to the results of the adjusted models above was seen, but with an even larger difference in sperm concentration and total sperm count between the men whose fathers were smokers and the men whose fathers were non-smokers (Table 4).

**Table 4:** Unadjusted mean values of semen parameters in relation to the paternal smoking in men of non-smoking mothers (cotinine < 15 ng/mL).

	Paternal smoking at the time of the pregnancy		
	Yes	No	P
	N = 17	N = 58^a^	
Sperm concentration (million/mL)^b^	**29**	**67**	**0.003**
Total sperm count (million) ^b^	**61**	**170**	**0.002**
Semen volume (mL)	2.3	2.7	0.25
Normal morphology (%)	7.1	9.3	0.15
Progressively motile (%)	53	53	0.96

^a^ 57 for sperm concentration and total sperm count

^b^ back-transformed from the cubic root

**Fig 2:**
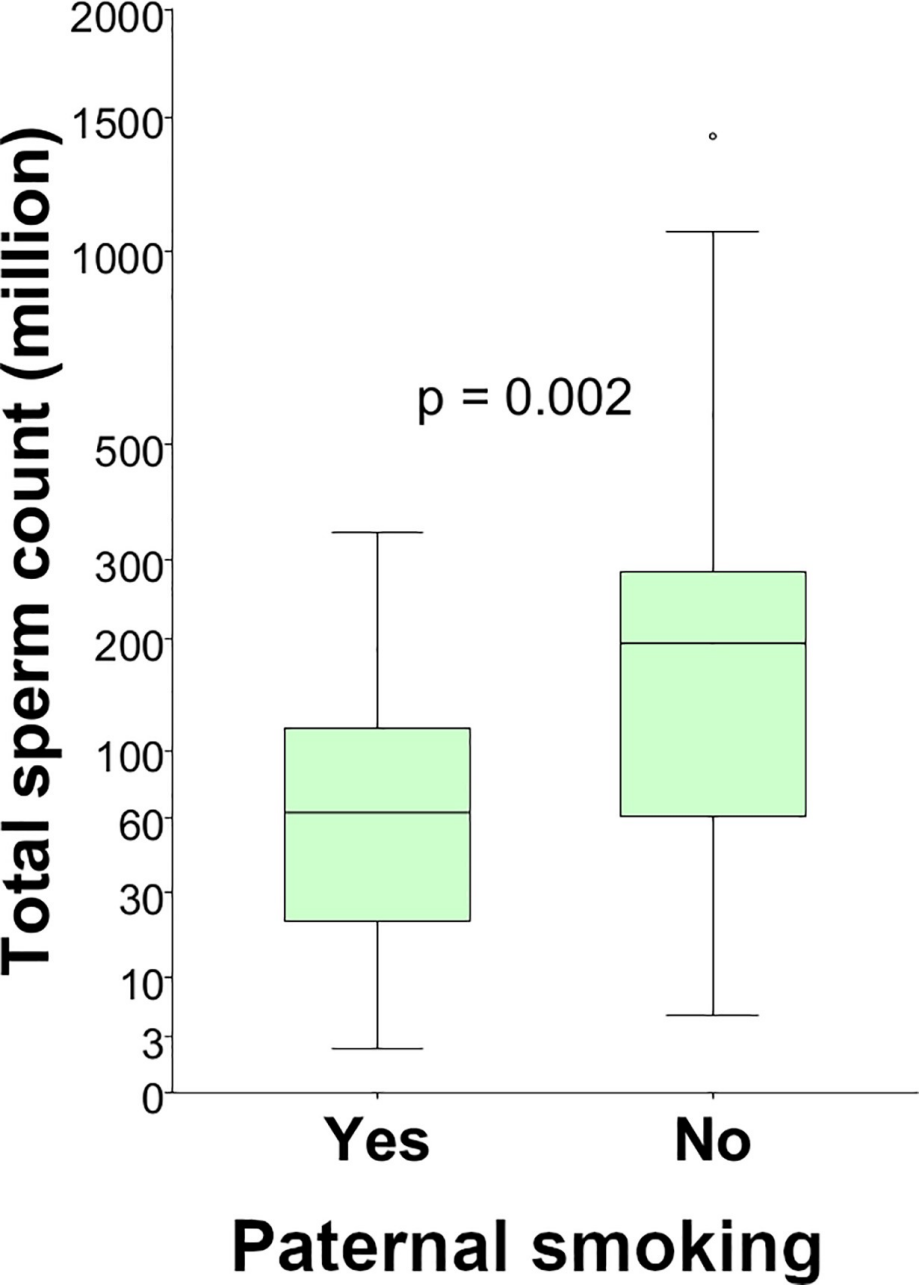
Total sperm counts in men of non-smoking mothers. The total sperm count in men of mothers that did not smoke (cotinine < 15ng/mL), divided in 17 men of whom the father smoked at the time of the pregnancy, and 57 men of whom the father did not. Dark lines in the middle of the boxes are median values. The bottom of the box indicates the 25^th^ percentile, and the top of the box indicates the 75^th^ percentile. The T-bars extend to 1.5 times the height of the box, or if less, to the minimum and maximum values. The circle signifies an outlier.

This difference was largest for the total sperm count (Fig 2) which was 64% lower in the 17 sons of smoking fathers as compared to the 57 sons of non-smoking fathers (61 x 10^6^ sperms vs 170 x 10^6^ sperms). For the other semen parameters, no association with paternal smoking was detected.

## Discussion

In this study, paternal smoking at the time of the pregnancy was associated with 40–50% lower sperm concentration and total sperm count in the sons. This finding is in line with our previous study [7], but now corroborated by adjusting for an objective estimate of maternal nicotine exposure, based on the levels of the metabolite cotinine in samples of serum obtained during the pregnancy. Our findings were also robust to adjustments for the men’s own smoking and to an additional adjustment for the estimated maternal socioeconomic status.

The major strength of this study is the access to maternal levels of cotinine, based on measurements in serum drawn predominantly early in the pregnancy. This measurement includes different modes of exposure, including both own smoking in the mothers and environmental exposure to tobacco smoke, such as through side-stream exposure from smoking fathers of our participants. Other general strengths include the homogeneity in the age and ethnicity of the participants, and the access to data on the maternal socioeconomic position.

A general weakness of the study is that the data regarding the paternal smoking were based only on questionnaires which were answered by the sons. Moreover, we had no data on the duration of the fathers’ smoking and neither of the number of cigarettes smoked per day. This may imply a risk of misclassification, and excludes the possibility to assess a possible dose-response relationship. Nevertheless, if paternal smoking was misclassified, this would rather have led to an underestimation of a true association or effect [31]. To continue, our study was rather limited in size, which requires a certain level of caution when the results are interpreted. Still, the limited study size could be regarded in view of the similar findings in our previous about three times larger study, which instead was based on data on maternal smoking from the Swedish Medical Birth Register [7]. In that study, the 104 men of the current study were also included. Thus, a similar result in the current study may therefore have been expected. However, if the lower sperm counts in paternally exposed men in the previous study were nevertheless due to an elevated maternal exposure to tobacco smoke (either as maternal smoking that was not registered in the Medical Birth Register, or as maternal environmental exposure), a disappearance of the association between paternal smoking and lower sperm counts could have been seen, given the ability to now fully adjust for the actual maternal exposure through any route. Therefore, the current study seems to indicate that the lower sperm counts in the paternally exposed men was unrelated to maternal levels of nicotine exposure, at least the specific time of sampling in the pregnancy. Still, although cotinine is a reliable marker of current smoking, it cannot be used in assessments of long-term exposure [32]. Therefore, we were not able to identify mothers who had recently stopped smoking, and may thus have underestimated maternal smoking taking place earlier in the pregnancy. Because of this, we can still not completely exclude that the men of the smoking fathers had decreased sperm counts due to a more pronounced maternal tobacco smoke exposure already prior to the sampling of the maternal serum. Still, the fact we in our previous study [7] did not find a lower total sperm count in men of smoking mothers than in men of non-smoking mothers, after the adjustment for paternal smoking, seems to argue against this as an explanation.

Most previous studies did not find any association between paternal smoking and semen quality in the sons [2–5]. However, these studies did not have access to a biomarker of maternal nicotine exposure during the pregnancy. Instead, those studies relied on data from questionnaires that were filled in by the mothers [2–4] or the sons [5]. To assess maternal smoking during pregnancy through questionnaires has been reported to be less reliable than measurements of cotinine since cotinine was reported more strongly negatively associated with birth weight [22]. Since the common coincidence of maternal and paternal smoking [11, 14] makes it difficult to separate potential effects from these two different parental exposures [11], the adjustment for maternal smoking in the studies above might have been inadequate when the association between paternal smoking and semen quality was studied. Furthermore, only two of the previous studies above seem to have investigated the specific association between paternal smoking and the total sperm count in the sons [2, 5]. Therefore, since the negative association with total sperm count was the strongest association we found, both in the current study and in our previous one [7], data rejecting an association between paternal smoking and total sperm count in sons could be regarded as somewhat limited.

The association we found does not prove a direct effect of paternal smoking since we cannot rule out other yet unidentified factors associated with both paternal smoking and lower sperm counts. Nonetheless, there seems to exist a clear evidence that smoking does induce sperm DNA damage and possibly also mutations in sperm [9]. Paternal smoking has, accordingly, been associated with DNA damage in the cord blood of the new-born child [33], germline mutations in repetitive DNA [34] and the *RB* gene [35], a shorter reproductive lifespan in daughters [8], different malformations in a majority of studies [9], and is considered as a cause of childhood cancer [10]. Although we did not specifically ask about paternal smoking taking place before the conception, paternal smoking before and during pregnancy appears to be correlated [11]. Therefore, these circumstances could together indicate that negative effects on sperm counts in the men of our study were transmitted via the father’s spermatozoa.

This topic needs further study before any stronger conclusions can be drawn. Nevertheless, in view of the other studies reporting associations between paternal smoking and various negative outcomes in the offspring, these different findings taken together seem to suggest that paternal smoking poses an underestimated environmental and life-style related hazard to the offspring. Further studies regarding smoking and potential effects on sperm DNA could possibly increase our understanding of possible mechanisms behind these findings. Such studies could benefit from taking also the consumption of alcohol into account, which in animal studies after paternal exposure before conception has been associated with different alterations in the offspring [36].

## Conclusions

Men of smoking fathers had about 50% lower total sperm count and sperm concentration than men of non-smoking fathers, also after adjustments for the levels of maternal exposure to nicotine during the pregnancy and the socioeconomic status.
